# The origin of the maximal lactate steady state (MLSS)

**DOI:** 10.1186/s13102-024-00827-3

**Published:** 2024-02-05

**Authors:** Hermann Heck, Henning Wackerhage

**Affiliations:** 1 Faculty for Sports Sciences, Ruhr Universität Bochum, Gesundheitscampus-Nord 10, 44801 Bochum, Germany; 2 Professorship for Exercise Biology, School of Medicine and Health, Technical University of Munich, Connollystraße 32, 80809 Munich, Germany

**Keywords:** Lactate, Graded exercise test, Maximal lactate steady state, MLSS

## Abstract

The maximal lactate steady state, abbreviated as MLSS, is the maximal exercise intensity where the concentration of earlobe capillary or arterial blood lactate remains constant over time. In the late 1970s and early 1980s, we (i.e. Hermann Heck and co-workers) developed a direct test to determine the MLSS to investigate whether it occurred at a lactate concentration of 4 mmol.L^− 1^, as earlier predicted by Alois Mader and colleagues. The test consisted of each participant performing several constant-intensity running bouts of ≈ 30 min at intensities close to the estimated MLSS. During each run, we measured lactate every 5 min. Based on the results, we defined the MLSS as the “*workload where the concentration of blood lactate does not increase more than 1 mmo*.*L*^*− 1*^*during the last 20 min of a constant load exercise*”. This MLSS protocol is impractical for performance testing as it requires too many exercise bouts, but it is a gold standard to determine the real MLSS. It is especially useful to validate indirect tests that seek to estimate the MLSS.

## Introduction

When humans exercise, the rate of muscular ATP hydrolysis increases up to 100-fold in the exercising muscles. As there are only around 5 mmol.L^− 1^ of ATP in skeletal muscle, ATP resynthesis must always closely match ATP hydrolysis as ATP depletion would lead to rigor mortis. ATP is resynthesized by phosphate transfer from phosphocreatine, by glycolysis, or by oxidative phosphorylation from carbohydrates or fat [1, 2]. As early as in the 1980s, Alois Mader has modelled this quantitatively and refined his model of human exercise metabolism in subsequent years [2–4].

Arguably the best blood biomarker for the state of energy metabolism during exercise is the arterial blood lactate concentration at a given exercise intensity. In relation to lactate, one phenomenon is the “maximal lactate steady state”. This is typically abbreviated as “maxLass” in German publications and “MLSS” in English publications. Other terms for the MLSS include “anaerobic threshold” ([5], “aerobic-anaerobic transition” [6] or “second lactate threshold (abbreviated as LT2)” [7]. The MLSS is, however, different (i.e. it occurs at a higher intensity) than the “aerobic threshold” or “first lactate threshold (abbreviated as LT1)” which is described as the point where the concentration of arterial lactate increases above baseline [7]. The MLSS occurs at the exercise intensity where metabolism changes from purely aerobic to partially anaerobic (see next paragraph about the use of the terms “*aerobic*” and “*anaerobic*”). The MLSS occurs also at the highest exercise intensity where the arterial lactate concentration remains constant over time, as all synthesized lactate can still be eliminated by oxidative phosphorylation and gluconeogenesis.

One important point is the use of the terms “*aerobic*” and “*anaerobic*”. Mader et al. use the term “*aerobic*” for oxidative phosphorylation, as it needs oxygen, and “*anaerobic*” for glycolysis with lactate synthesis, as it does not require oxygen. They do not use the term “*anaerobic*” to indicate hypoxia or to suggest that hypoxia is a critical activator of glycolytic flux [8]. The fact that hypoxia is not required for glycolytic flux and lactate synthesis is known since Otto Warburg’s seminal experiments on cancer cells [9]. Mader et al. clearly appreciate this as is evident from the equation that models glycolytic flux in Mader’s mathematical model of human exercise metabolism. This equation does not feature oxygen as a regulator [2, 4].

The MLSS testing protocol of performing repeated 30-min exercise bouts near the suspected MLSS is the gold standard protocol to determine the MLSS, as it directly determines the highest constant intensity where the concentration of lactate remains constant in blood over time. Importantly, the MLSS describes the maximal steady state of blood lactate and not the maximal metabolic steady state [10].

Intriguingly, the origin of the MLSS and the MLSS testing protocol is unclear or unknown especially in the English-speaking world. For example, Jones and colleagues state “*The origin of the MLSS concept is somewhat obscure but it may perhaps be attributed to the work of German physiologists, Mader and Heck, in the 1980s”* [10]. Moreover, a 30-page review in the Journal of Physiology entitled “*the anaerobic threshold: 50 + years of controversy*” [11] failed to mention the MLSS entirely, and thereby contributed to the controversy. Given that the origin of the MLSS is perceived as “*somewhat obscure*” and not acknowledged as being part of 50 years of anaerobic threshold research, we aim to describe the ideas and experiments that led to the formulation of the MLSS by us (i.e. Hermann Heck and co-workers). In this review, we first describe the ideas and experiments that led to the concept of the MLSS and then recommend tests to directly measure the MLSS or to indirectly estimate it with a running and cycling test.

### Work that led to the MLSS

In 1976, Alois Mader and colleagues of the Cologne group of Wildor Hollmann published a paper in German where they introduced a first lactate threshold or transition that marks the “*exercise intensity where muscle metabolism changes from purely aerobic to partially anaerobic*”. Mader, our co-authors and myself estimated that this transition occurred at an earlobe capillary blood lactate concentration of 4 mmol.L^− 1^ (the earlobe capillary blood lactate concentration is comparable to the lactate concentration in arterial blood). Specifically, we stated *“Up to an intensity that causes a lactate concentration of 4 mmol*.*L*^*− 1*^*in the blood, the concentration of lactate remains constant or decreases when continuing the given load as a sign of an oxidative energy balance. At higher intensities with blood lactate concentrations above 5.0 mmol*.*L*^*− 1*^*after the first minutes of exercise, the concentration of lactate continues to rise as an expression of a permanent energy deficit that is then covered by lactate synthesis”* [6].

One limitation of our 1976 publication [6] was that we did not show data to support the claim that the “*aerobic-anaerobic transition*” (today also termed “anaerobic threshold”, “second lactate threshold” (abbreviated as LT2) or “onset of blood lactate accumulation” (abbreviated as OBLA) [7]) occurred at an exercise intensity where the lactate concentration is 4 mmol.L^− 1^. Some researchers reasoned that the lactate concentration at the intensity where lactate started to increase over time varied inter-individually and they termed this the individual lactate threshold. Several groups then used graphical or mathematical methods as well as other fixed lactate concentrations to estimate the individual lactate threshold. The methods used to determine the individual lactate threshold include the following:

1. Determination of the point where a tangent touches the lactate curve [12].
2. Determination of the point where a tangent touches the lactate curve [13] by a different method than in [12].
3. Determination of the point where the angle bisector touches that lactate curve [14].
4. Intensity at 1.5-mmol-L^-1^ above the lowest lactate concentration [15].
5. Dmax method, i.e. the power with the greatest distance between a line between the lowest and highest lactate and the lactate curve [16].

The obvious problem with these approaches was, however, that these indirect estimates of the MLSS where not compared against a directly measured MLSS as a gold standard. For a more detailed description of lactate threshold concepts see relevant reviews [17, 18] and a 652-page book on lactate in German by myself and my colleagues [19].

**Fig. 1:**
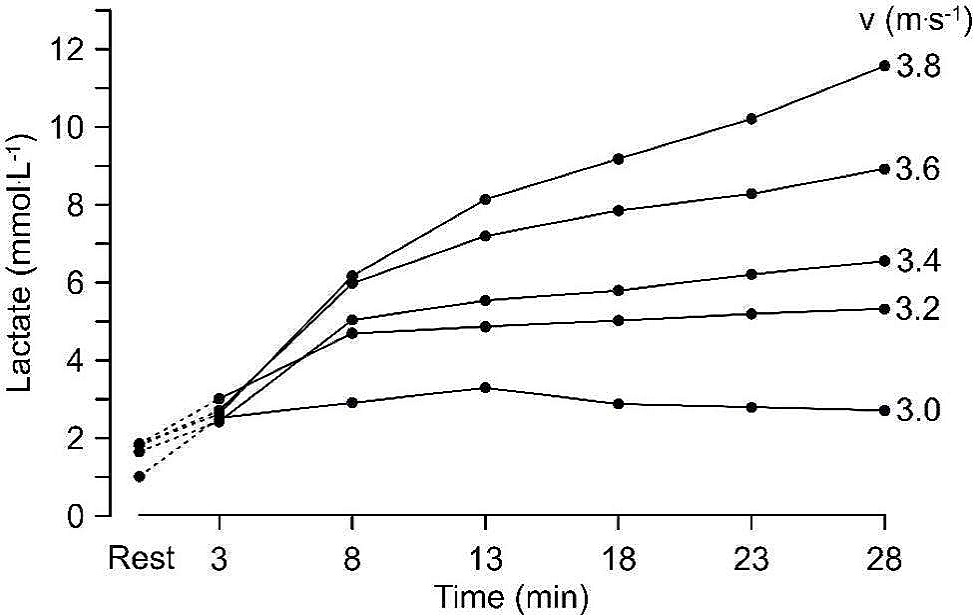
Concentration of lactate during 5 constant speed running trials near the MLSS performed by one participant. For each trial, the participant warmed up for 3 min with approximately 70% of the subsequent constant speed run before running at in-between 3.0 and 3.8 m/s for 25 min [20]

In the late 1970s, I (i.e. Hermann Heck) and my colleagues at the time started to conduct experiments that aimed to answer the question “Does the aerobic-anaerobic transition (i.e., MLSS) occur at a blood lactate concentration of 4 mmol/L as hypothesized by Mader et al [6]?” To answer this question, we conducted experiments aimed at directly identifying the maximal exercise intensity where the blood lactate concentration remained constant. We realised that our participants needed to conduct several constant load exercise trials to identify the intensity where lactate started to rise over time. Starting in 1979, I and my colleagues conducted the first experiments involving repeated constant velocity runs on a treadmill. After a 3-minute warm up with 70% of the subsequent running intensity, participants ran for 25 min at a fixed exercise intensity. After each 5 min, the participants stopped for 30 s for ear lobe blood sampling and lactate measurement, a method established earlier Alois Mader [3]. Figure 1 shows a typical example for one subject during five such 25 min running trials with velocities from 3.0 to 3.8 m.s^− 1^.

**Fig. 2:**
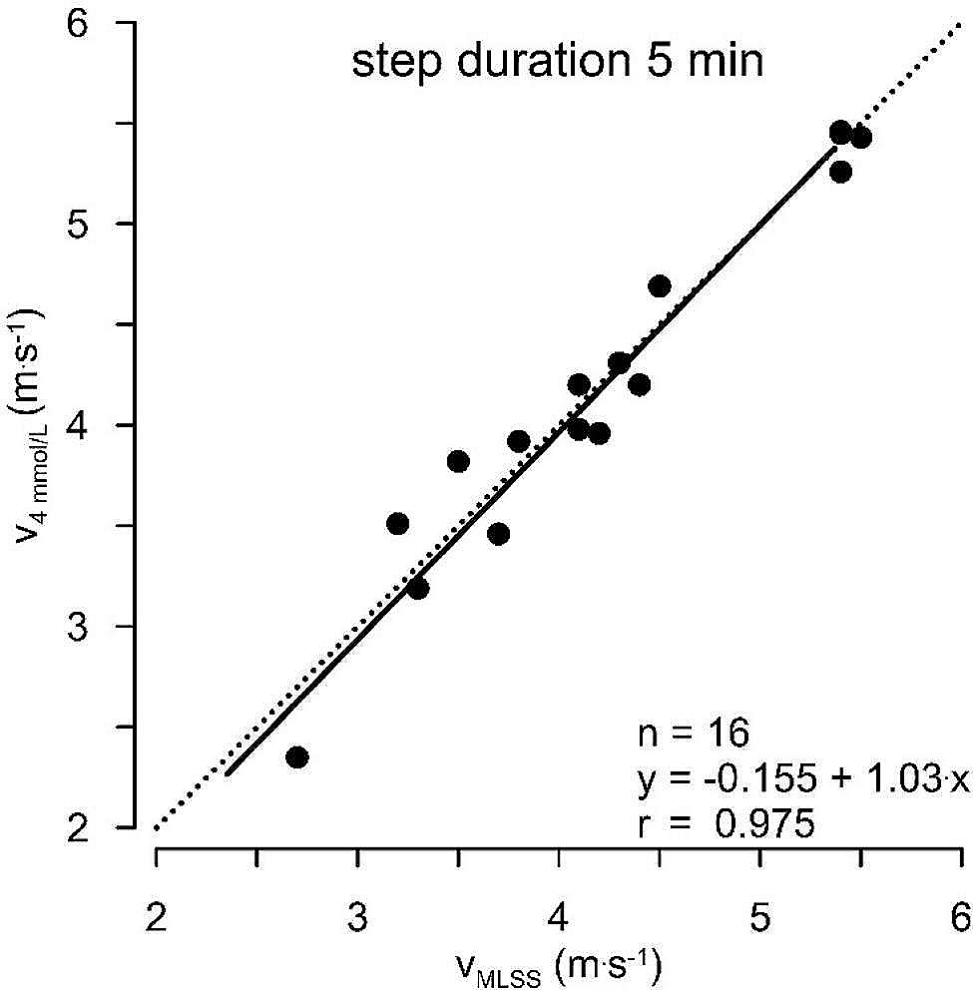
Regression between the velocity where the MLSS is reached and the velocity where 4 mmol.L^− 1^ are reached in a graded running test on the treadmill with 5 min steps and increase of 0.4 m.s^− 1^ per step [21]

These experiments on 15 participants revealed to us that the lactate concentration remained constant over time at intensities where the participants reached a blood lactate concentration of 4.0 ± 0.7 mmol/L from the 10th to the 25th minute of the trial (see Fig. 2).

These experiments confirmed the assumption by Mader et al. [6] that the transition from aerobic to partially anaerobic metabolism occurs on average at approximately 4 mmol/l of lactate. In 1982, we published this data in German and used the German term “*maximales Laktatgleichgewicht*” [20].

In one German [22] and one English [23] publication in 1985, I then used the term “*maximal lactate steady state*” (the German term “*Gleichgewicht*” is “*steady state*” in English) which marks the first usage of the English term. This term is typically abbreviated as “*maxLass*” in German author publications and “*MLSS*” in English author publications. Our definition of the MLSS is the “*workload where the concentration of blood lactate does not increase more than 1 mmol*.*L*^*− 1*^*during the last 20 min of a constant load exercise*” [23].

In the 1970s and early 1980s, American researchers used the term “*maximal steady state*” in some of their publications, too [24, 25]. Because of the similarity with the term MLSS we compare the ideas behind these terms. In their publications, the American researchers realised like Mader et al. (1976) that the V̇O_2_max was a poor biomarker for the level of conditioning and said that “*no test presently exists which can differentiate levels of conditioning*”. Because of that, they did experiments to identify a better biomarker or test for the level of conditioning. They found that endurance athletes could exercise at a higher relative intensity than sedentary participants before the plasma lactate concentration started to *“accumulate at a rapid rate”*. Based on their research they stated that the maximal steady state was an intensity at an arbitrarily chosen plasma (i.e. venous) lactate concentration of 2.2 mmol.L^−1^ (roughly equivalent to an earlobe capillary or arterial concentration of ≈ 2.9 mmol.L^−1^) and that the intensity at this maximal steady state was a better measure for the state of conditioning than the V̇O_2_max [24, 25]. So, was this “*maximal steady state*” identical to the MLSS? Whilst both groups realised that there was a maximal intensity before the concentration of lactate was rising over time, it was only us who conducted constant load experiments with repeated lactate measurements to identify the maximal running speed or power where lactate did not rise over time. Moreover, we (Heck et al. 1985) stated that this was the point of a “*maximal balance between lactate production and elimination*” [23]. Thus, the maximal steady state at a plasma lactate concentration of 2.2 mmol.L^−1^ of Londeree and Ames (1975) was primarily seen as a measure for the conditioning of an athlete and not as a test protocol to identify the maximal power or speed where all synthesized lactate could still be eliminated and where the concentration of lactate remained still constant over time.

### Effect of the test protocol on the lactate concentration where the MLSS occurs in a graded exercise test

Already in the 1970s, the Cologne group realised that the test protocol influenced the relationship in-between workload and blood lactate concentration. They stated “*For the reasons mentioned above, the duration of work should therefore not fall below 4 minutes per step for an objective assessment of endurance performance. It is better to select a duration of between 5 and 10 minutes for each step*” [6]. Generally, the steeper the increase of power or speed per minute in a continuous or stepwise graded exercise test protocol, the lower the blood lactate where the MLSS intensity is reached. The mechanism is that the blood lactate concentration must reach a steady state at intensities below the MLSS and because above the MLSS lactate increases over time. So, if the time at a given power or speed is shorter, then the blood lactate concentration will be lower.

**Fig. 3:**
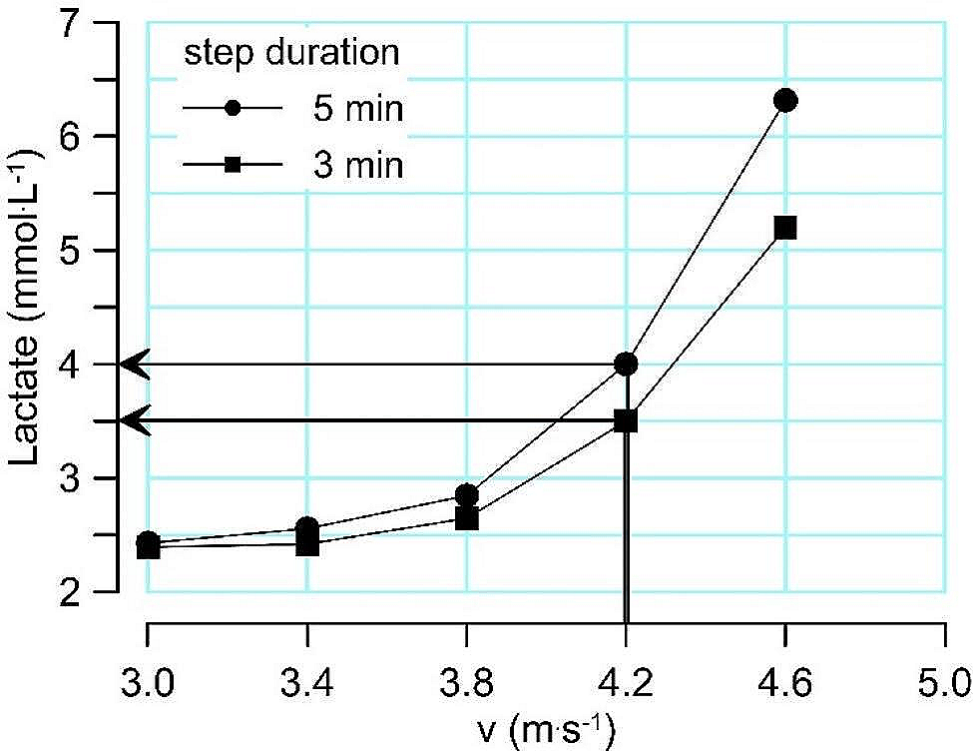
Example for how reducing the step duration from 5 min to 3 min per increase of 0.4 m.s^− 1^ results in a drop of the blood lactate concentration where the MLSS velocity of 4.2 m.s^− 1^ is reached

After establishing the MLSS testing protocol, I and my colleagues performed graded exercise tests where we investigated the effect of the rate of increase of power (W) or speed per minute on the blood lactate concentration where the MLSS occurs (i.e., the power or running speed at MLSS). We found that when we reduced the step duration of a graded cycling test from 5 min to 3 min, then the blood lactate concentration where the participants reached the MLSS power dropped on average from 4 to 3.5 mmol/L. Figure 3 shows an example of this drop in the lactate concentration during a graded running test.

**Fig. 4:**
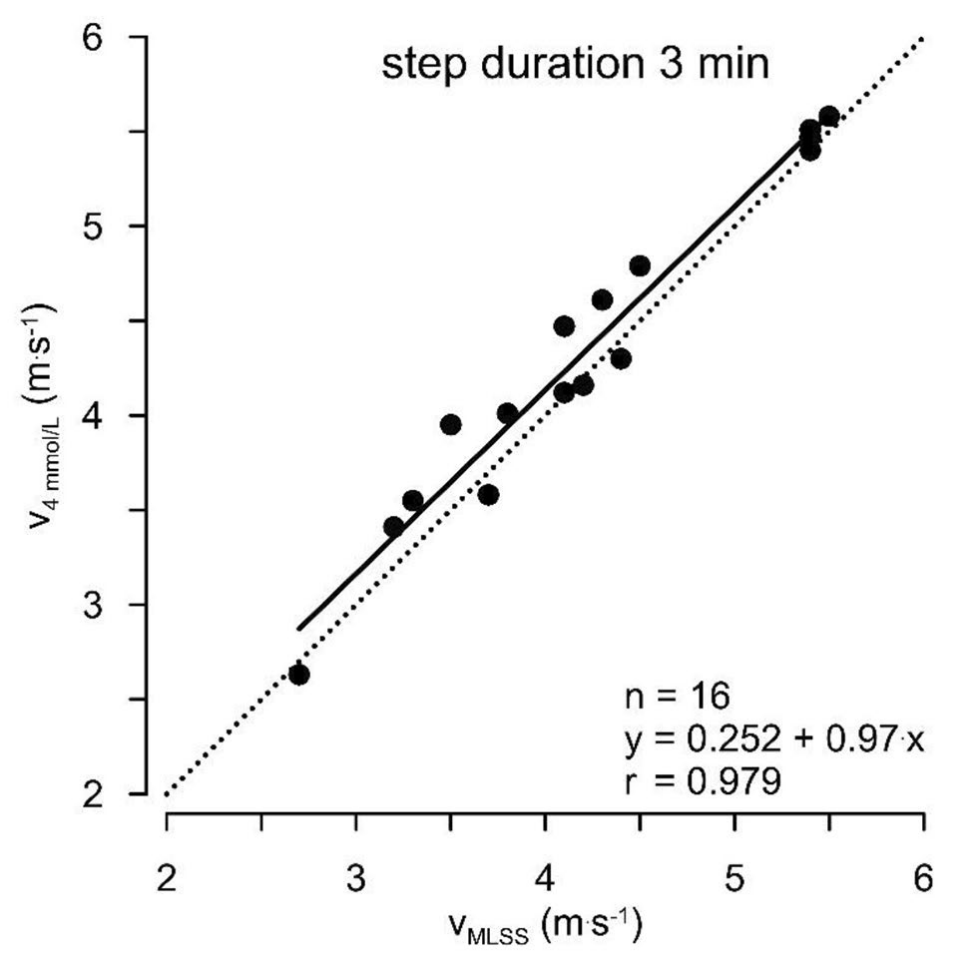
shows for 15 participants the correlation between blood lactate concentration in a test where the running velocity increased by 0.4 m.s^− 1^ all 3 min with the running velocity where the MLSS is reached

Figure 4. Correlation between the running velocity where the MLSS is reached and between the running velocity where a blood lactate concentration of 4 mmol.L^-1^ is reached in a graded running test. The experimenters increased running velocity by 0.4 m.s^− 1^ every 3 min. v_4mmol/L_ is the velocity at 4 mmol/L of lactate and v_MLSS_ the velocity at the maximal lactate steady state.

I published the results of these experiments in my habilitation (i.e., a dissertation and degree that qualifies for professorship in Germany), and 1990 as a book in German. Additionally, we conducted experiments to study the effect of different test protocols on blood lactate concentrations during graded cycle ergometry tests (see Table 1).

**Table 1:** Tests to investigate the effect of the increase of power during cycle ergometer tests (published on pages 156–171 in [21])

Test	Starting Power	Power increment	Step duration	Power increase per unit of time	Typical usage of test
A	50 W	50 W	2 min	25.0 W.min^− 1^	Rowers and cyclists
B	50 W	50 W	3 min	16.7 W.min^− 1^	Commonly used in Germany
C	25 W	25 W	2 min	12.5 W.min^− 1^	Untrained participants, children, patients
D	70, 100 or 130 W	30 W	5 min	6 W.min^− 1^	Only used for this experiment

**Fig. 5:**
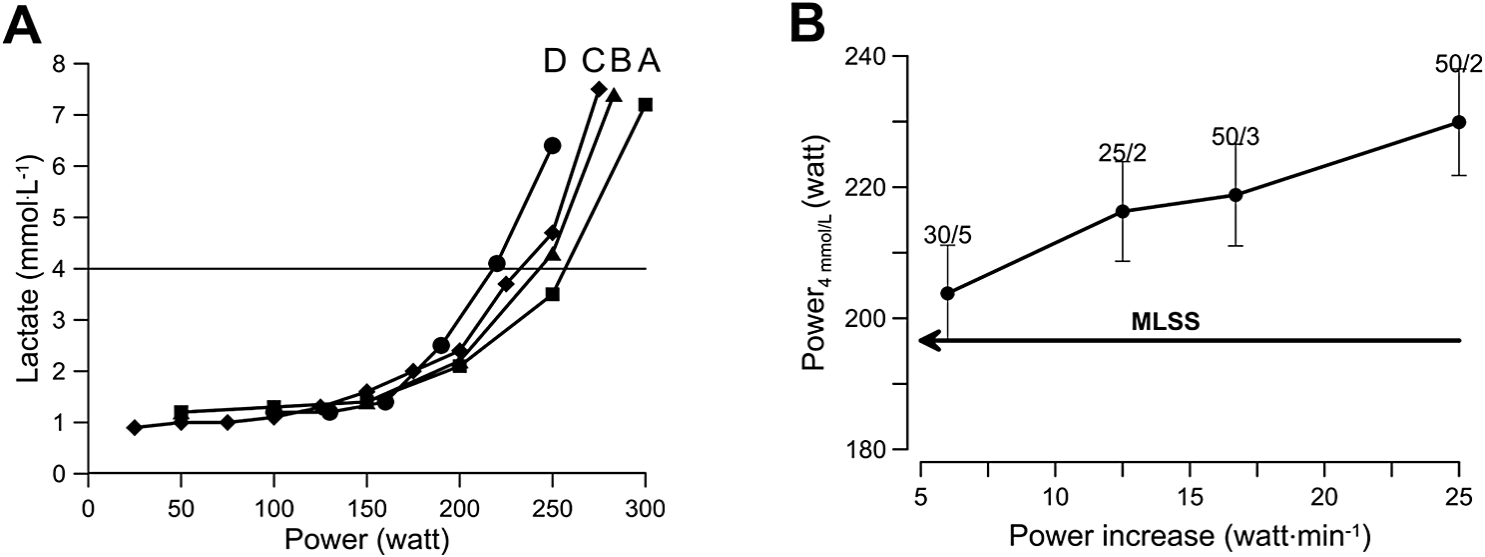
**A** Example of lactate curves of a participant who has performed all four tests that are listed in Table 1. Note that the steeper the increase of power over time, the higher the workload where a lactate concentration of 4 mmol.L^− 1^ is reached. **B** Overall data showing how the power increase per unit of time affects the power where a blood lactate concentration of 4 mmol.L^− 1^ is reached. Above each point x/y is given whereby x is the increase of power per step in W and where y is the duration of each step in minutes

The results of the experiments shown in Table 1 are illustrated in Fig. 5. Figure 5A shows the results for one individual and Fig. 5B illustrates how the increase in power over unit of time affects the power where a blood lactate concentration of 4 mmol.L^− 1^ is reached.

**Fig. 6:**
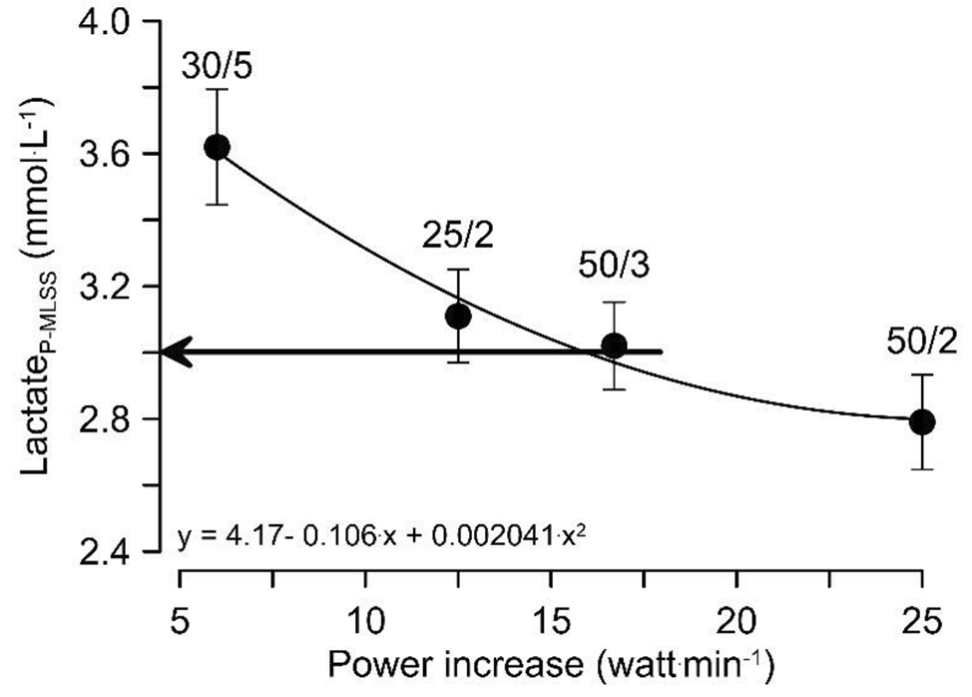
Relationship between the increase of power (e.g., 50/3 means increasing power by 50 W all 3 min) and the lactate concentration where the MLSS power is reached (see Heck et al. 2022, S.371)

In Germany and elsewhere, a common graded cycle exercise test is to increase the work load all 3 min by 50 W. In this test, the MLSS power (i.e. the maximal constant load power where blood lactate remains constant) is reached on average at approximately 3 mmol.L^− 1^ of lactate (see Fig. 6).

## Summary and conclusions

The MLSS method is the only direct test to measure the MLSS. However, it is impractical for performance testing as it requires several ≈ 30 min long exercise bouts to determine it. It is, however, a gold standard testing protocol to validate indirect tests that seek to estimate the MLSS.

To estimate the MLSS during cycle ergometry, we recommend a graded exercise test where the starting load is based on fitness and where power is then increased by 50 W every 3 min. In this test, the power at a lactate concentration of 3 mmol.L^− 1^ approximately predicts the power at the MLSS in the direct MLSS protocol.

## Data Availability

This is a review that contains some data generated decades ago e.g., during experiments for students’ theses. We aim to make this data available in response to requests.

## Abbreviations

MLSS: Maximal lactate steady state
v_4mmol/L_: Velocity at 4 mmol/L of lactate
v_MLSS_: Velocity at the maximal lactate steady state
